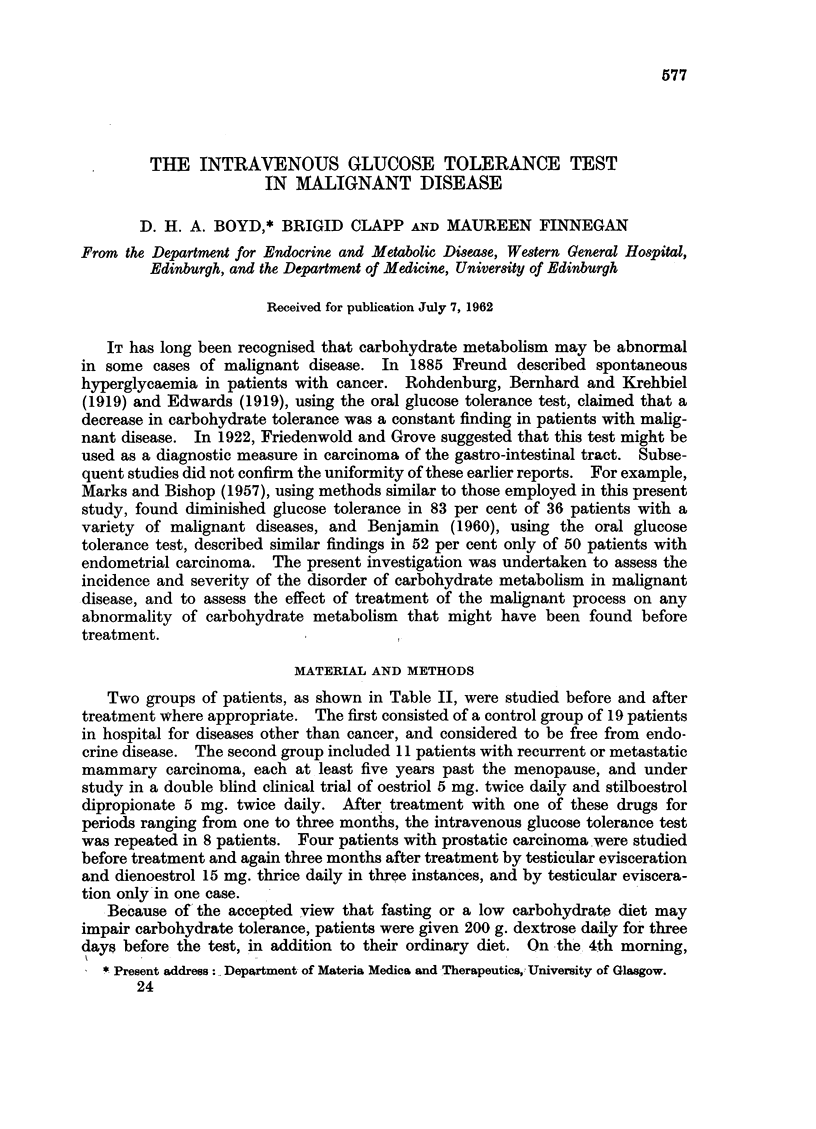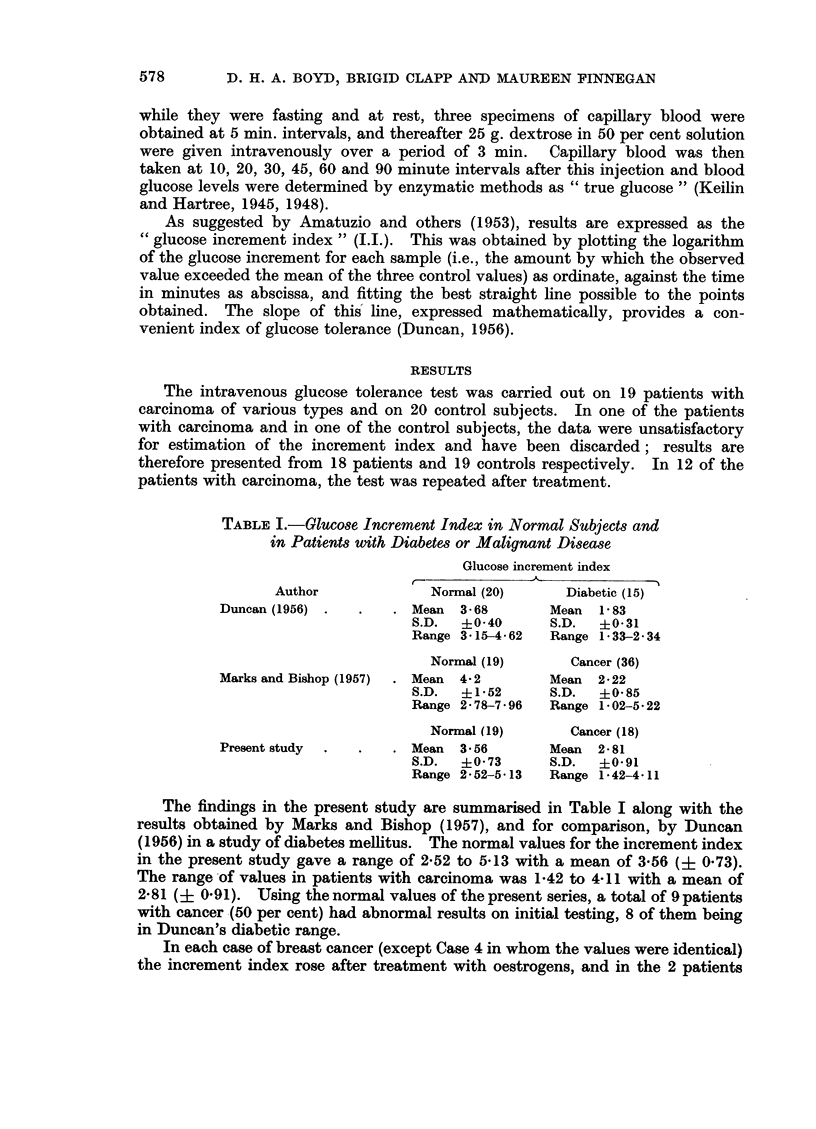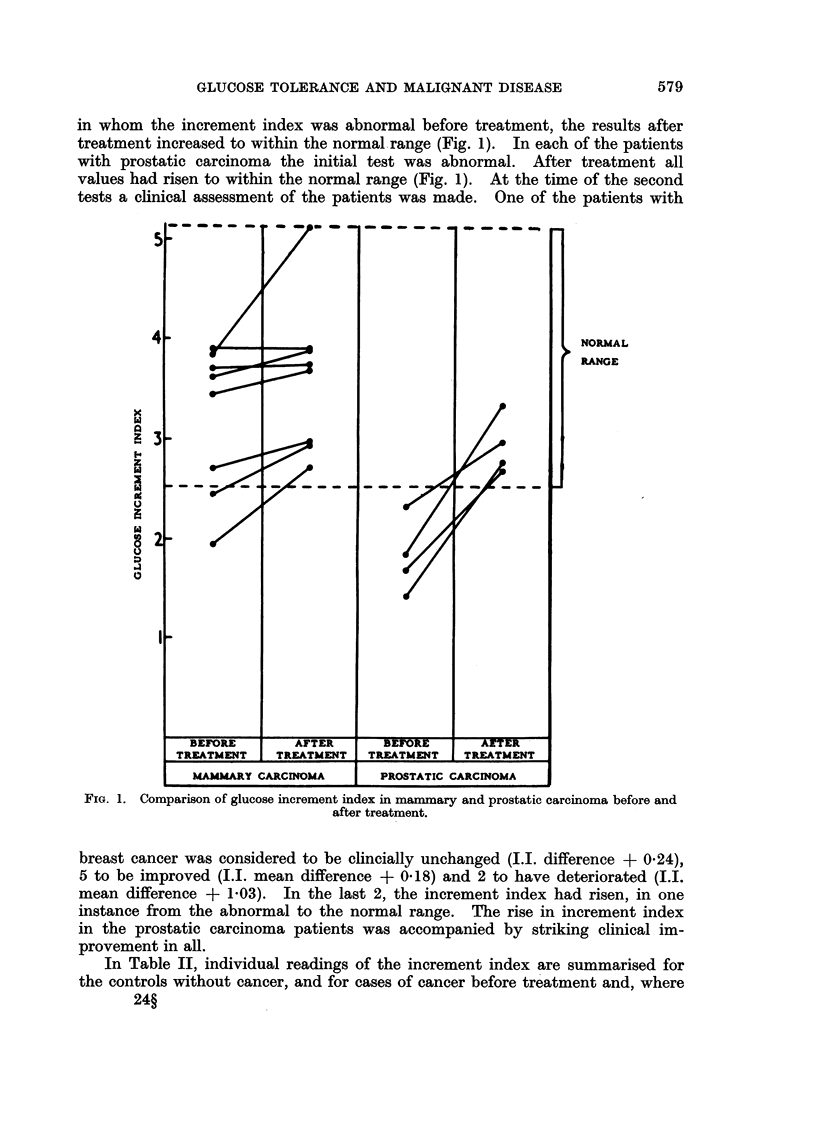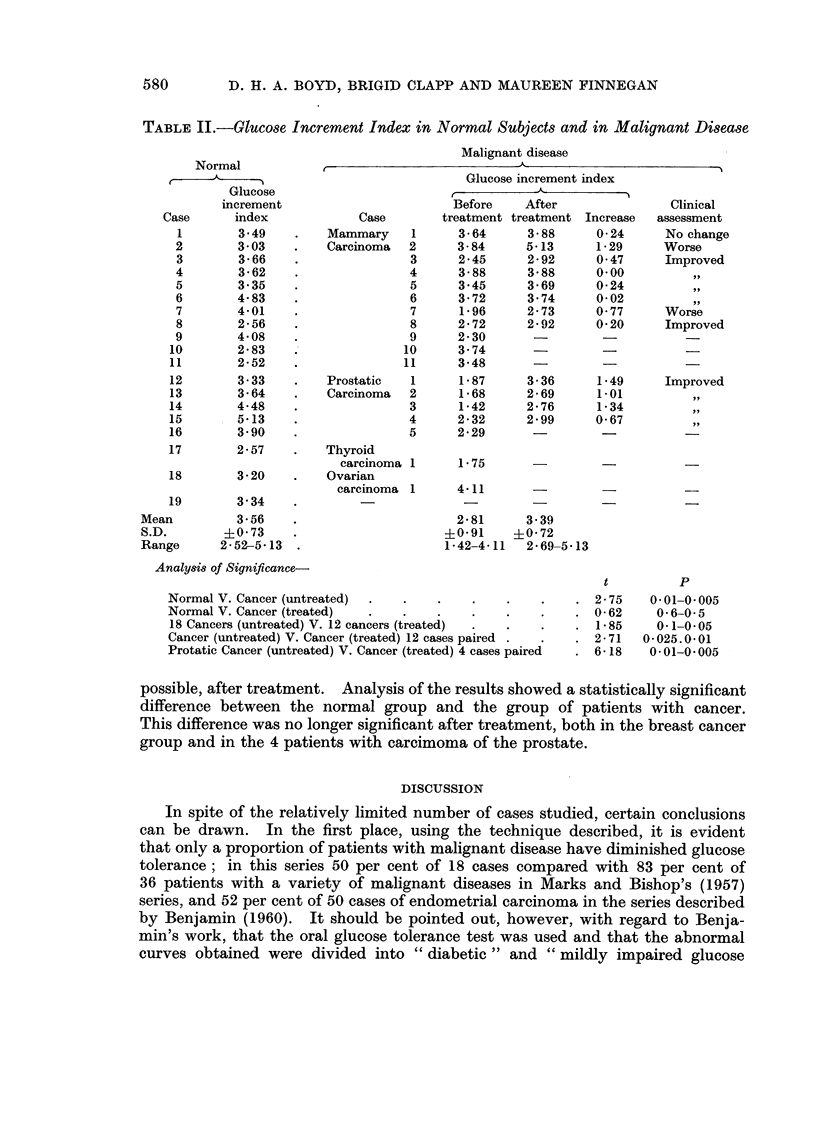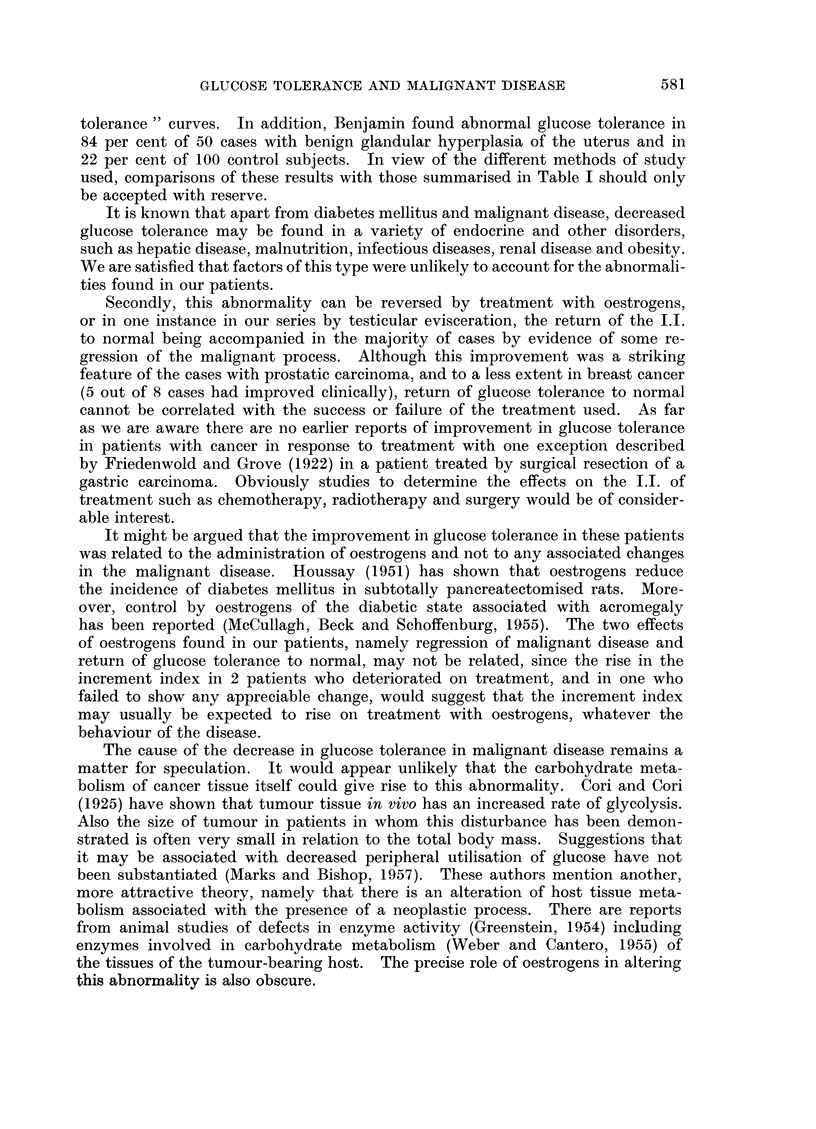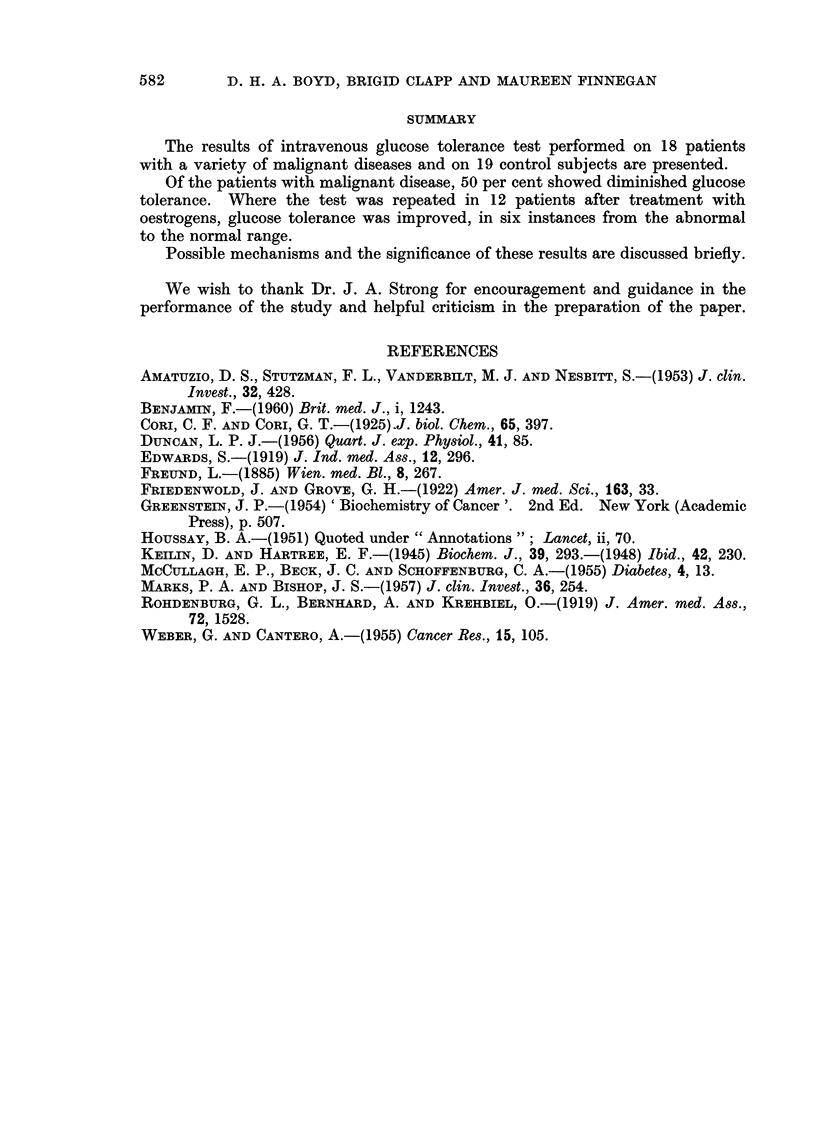# The Intravenous Glucose Tolerance Test in Malignant Disease

**DOI:** 10.1038/bjc.1962.67

**Published:** 1962-09

**Authors:** D. H. A. Boyd, Brigid Clapp, Maureen Finnegan


### THE INTRAVENOUS GLUCOSE TOLERANCE TEST IN MALIGNANT DISEASE

D. H. A. BOYD,* BRIGID CLAPP AND MAUREEN FINNEGAN

*From the Department for Endocrine and Metabolic Disease, Western General Hospital, Edinburgh, and the Department of Medicine, University of Edinburgh*

Received for publication July 7, 1962

It has long been recognised that carbohydrate metabolism may be abnormal in some cases of malignant disease. In 1885 Freund described spontaneous hyperglycaemia in patients with cancer. Rohdenburg, Bernhard and Krehbiel (1919) and Edwards (1919), using the oral glucose tolerance test, claimed that a decrease in carbohydrate tolerance was a constant finding in patients with malignant disease. In 1922, Friedenwold and Grove suggested that this test might be used as a diagnostic measure in carcinoma of the gastro-intestinal tract. Subsequent studies did not confirm the uniformity of these earlier reports. For example, Marks and Bishop (1957), using methods similar to those employed in this present study, found diminished glucose tolerance in 83 per cent of 36 patients with a variety of malignant diseases, and Benjamin (1960), using the oral glucose tolerance test, described similar findings in 52 per cent only of 50 patients with endometrial carcinoma. The present investigation was undertaken to assess the incidence and severity of the disorder of carbohydrate metabolism in malignant disease, and to assess the effect of treatment of the malignant process on any abnormality of carbohydrate metabolism that might have been found before treatment.

### MATERIAL AND METHODS

Two groups of patients, as shown in Table II, were studied before and after treatment where appropriate. The first consisted of a control group of 19 patients in hospital for diseases other than cancer, and considered to be free from endocrine disease. The second group included 11 patients with recurrent or metastatic mammary carcinoma, each at least five years past the menopause, and under study in a double blind clinical trial of oestriol 5 mg. twice daily and stilboestrol dipropionate 5 mg. twice daily. After treatment with one of these drugs for periods ranging from one to three months, the intravenous glucose tolerance test was repeated in 8 patients. Four patients with prostatic carcinoma were studied before treatment and again three months after treatment by testicular evisceration and dienoestrol 15 mg. thrice daily in three instances, and by testicular evisceration only in one case.

Because of the accepted view that fasting or a low carbohydrate diet may impair carbohydrate tolerance, patients were given 200 g. dextrose daily for three days before the test, in addition to their ordinary diet. On the 4th morning,

* Present address: Department of Materia Medica and Therapeutics, University of Glasgow.

while they were fasting and at rest, three specimens of capillary blood were obtained at 5 min. intervals, and thereafter 25 g. dextrose in 50 per cent solution were given intravenously over a period of 3 min. Capillary blood was then taken at 10, 20, 30, 45, 60 and 90 minute intervals after this injection and blood glucose levels were determined by enzymatic methods as " true glucose " (Keilin and Hartree, 1945, 1948).

As suggested by Amatuzio and others (1953), results are expressed as the " glucose increment index " (I.I.). This was obtained by plotting the logarithm of the glucose increment for each sample (i.e., the amount by which the observed value exceeded the mean of the three control values) as ordinate, against the time in minutes as abscissa, and fitting the best straight line possible to the points obtained. The slope of this line, expressed mathematically, provides a convenient index of glucose tolerance (Duncan, 1956).

### RESULTS

The intravenous glucose tolerance test was carried out on 19 patients with carcinoma of various types and on 20 control subjects. In one of the patients with carcinoma and in one of the control subjects, the data were unsatisfactory for estimation of the increment index and have been discarded; results are therefore presented from 18 patients and 19 controls respectively. In 12 of the patients with carcinoma, the test was repeated after treatment.

TABLE I.—*Glucose Increment Index in Normal Subjects and in Patients with Diabetes or Malignant Disease*

| Author | Glucose increment index | | | |
|---|---|---|---|---|
| | Normal (20) | | Diabetic (15) | |
| Duncan (1956) | Mean | 3·68 | Mean | 1·83 |
| | S.D. | ±0·40 | S.D. | ±0·31 |
| | Range | 3·15–4·62 | Range | 1·33–2·34 |
| | Normal (19) | | Cancer (36) | |
| Marks and Bishop (1957) | Mean | 4·2 | Mean | 2·22 |
| | S.D. | ±1·52 | S.D. | ±0·85 |
| | Range | 2·78–7·96 | Range | 1·02–5·22 |
| | Normal (19) | | Cancer (18) | |
| Present study | Mean | 3·56 | Mean | 2·81 |
| | S.D. | ±0·73 | S.D. | ±0·91 |
| | Range | 2·52–5·13 | Range | 1·42–4·11 |

The findings in the present study are summarised in Table I along with the results obtained by Marks and Bishop (1957), and for comparison, by Duncan (1956) in a study of diabetes mellitus. The normal values for the increment index in the present study gave a range of 2·52 to 5·13 with a mean of 3·56 (± 0·73). The range of values in patients with carcinoma was 1·42 to 4·11 with a mean of 2·81 (± 0·91). Using the normal values of the present series, a total of 9 patients with cancer (50 per cent) had abnormal results on initial testing, 8 of them being in Duncan's diabetic range.

In each case of breast cancer (except Case 4 in whom the values were identical) the increment index rose after treatment with oestrogens, and in the 2 patients in whom the increment index was abnormal before treatment, the results after treatment increased to within the normal range (Fig. 1). In each of the patients with prostatic carcinoma the initial test was abnormal. After treatment all values had risen to within the normal range (Fig. 1). At the time of the second tests a clinical assessment of the patients was made. One of the patients with

FIG. 1. Comparison of glucose increment index in mammary and prostatic carcinoma before and after treatment.

breast cancer was considered to be clincially unchanged (I.I. difference + 0·24), 5 to be improved (I.I. mean difference + 0·18) and 2 to have deteriorated (I.I. mean difference + 1·03). In the last 2, the increment index had risen, in one instance from the abnormal to the normal range. The rise in increment index in the prostatic carcinoma patients was accompanied by striking clinical improvement in all.

In Table II, individual readings of the increment index are summarised for the controls without cancer, and for cases of cancer before treatment and, where

TABLE II.—*Glucose Increment Index in Normal Subjects and in Malignant Disease*

| Normal | | Malignant disease | | | | | |
|---|---|---|---|---|---|---|---|
| | | | | Glucose increment index | | | |
| Case | Glucose increment index | Case | | Before treatment | After treatment | Increase | Clinical assessment |
| 1 | 3·49 | Mammary | 1 | 3·64 | 3·88 | 0·24 | No change |
| 2 | 3·03 | Carcinoma | 2 | 3·84 | 5·13 | 1·29 | Worse |
| 3 | 3·66 | | 3 | 2·45 | 2·92 | 0·47 | Improved |
| 4 | 3·62 | | 4 | 3·88 | 3·88 | 0·00 | ,, |
| 5 | 3·35 | | 5 | 3·45 | 3·69 | 0·24 | ,, |
| 6 | 4·83 | | 6 | 3·72 | 3·74 | 0·02 | ,, |
| 7 | 4·01 | | 7 | 1·96 | 2·73 | 0·77 | Worse |
| 8 | 2·56 | | 8 | 2·72 | 2·92 | 0·20 | Improved |
| 9 | 4·08 | | 9 | 2·30 | — | — | — |
| 10 | 2·83 | | 10 | 3·74 | — | — | — |
| 11 | 2·52 | | 11 | 3·48 | — | — | — |
| 12 | 3·33 | Prostatic | 1 | 1·87 | 3·36 | 1·49 | Improved |
| 13 | 3·64 | Carcinoma | 2 | 1·68 | 2·69 | 1·01 | ,, |
| 14 | 4·48 | | 3 | 1·42 | 2·76 | 1·34 | ,, |
| 15 | 5·13 | | 4 | 2·32 | 2·99 | 0·67 | ,, |
| 16 | 3·90 | | 5 | 2·29 | — | — | — |
| 17 | 2·57 | Thyroid carcinoma | 1 | 1·75 | — | — | — |
| 18 | 3·20 | Ovarian carcinoma | 1 | 4·11 | — | — | — |
| 19 | 3·34 | — | | — | — | — | — |
| Mean | 3·56 | | | 2·81 | 3·39 | | |
| S.D. | ±0·73 | | | ±0·91 | ±0·72 | | |
| Range | 2·52–5·13 | | | 1·42–4·11 | 2·69–5·13 | | |

*Analysis of Significance*—

| | *t* | *P* |
|---|---|---|
| Normal V. Cancer (untreated) | 2·75 | 0·01–0·005 |
| Normal V. Cancer (treated) | 0·62 | 0·6–0·5 |
| 18 Cancers (untreated) V. 12 cancers (treated) | 1·85 | 0·1–0·05 |
| Cancer (untreated) V. Cancer (treated) 12 cases paired | 2·71 | 0·025.0·01 |
| Protatic Cancer (untreated) V. Cancer (treated) 4 cases paired | 6·18 | 0·01–0·005 |

possible, after treatment. Analysis of the results showed a statistically significant difference between the normal group and the group of patients with cancer. This difference was no longer significant after treatment, both in the breast cancer group and in the 4 patients with carcimoma of the prostate.

### DISCUSSION

In spite of the relatively limited number of cases studied, certain conclusions can be drawn. In the first place, using the technique described, it is evident that only a proportion of patients with malignant disease have diminished glucose tolerance ; in this series 50 per cent of 18 cases compared with 83 per cent of 36 patients with a variety of malignant diseases in Marks and Bishop's (1957) series, and 52 per cent of 50 cases of endometrial carcinoma in the series described by Benjamin (1960). It should be pointed out, however, with regard to Benjamin's work, that the oral glucose tolerance test was used and that the abnormal curves obtained were divided into " diabetic " and " mildly impaired glucose tolerance " curves. In addition, Benjamin found abnormal glucose tolerance in 84 per cent of 50 cases with benign glandular hyperplasia of the uterus and in 22 per cent of 100 control subjects. In view of the different methods of study used, comparisons of these results with those summarised in Table I should only be accepted with reserve.

It is known that apart from diabetes mellitus and malignant disease, decreased glucose tolerance may be found in a variety of endocrine and other disorders, such as hepatic disease, malnutrition, infectious diseases, renal disease and obesity. We are satisfied that factors of this type were unlikely to account for the abnormalities found in our patients.

Secondly, this abnormality can be reversed by treatment with oestrogens, or in one instance in our series by testicular evisceration, the return of the I.I. to normal being accompanied in the majority of cases by evidence of some regression of the malignant process. Although this improvement was a striking feature of the cases with prostatic carcinoma, and to a less extent in breast cancer (5 out of 8 cases had improved clinically), return of glucose tolerance to normal cannot be correlated with the success or failure of the treatment used. As far as we are aware there are no earlier reports of improvement in glucose tolerance in patients with cancer in response to treatment with one exception described by Friedenwold and Grove (1922) in a patient treated by surgical resection of a gastric carcinoma. Obviously studies to determine the effects on the I.I. of treatment such as chemotherapy, radiotherapy and surgery would be of considerable interest.

It might be argued that the improvement in glucose tolerance in these patients was related to the administration of oestrogens and not to any associated changes in the malignant disease. Houssay (1951) has shown that oestrogens reduce the incidence of diabetes mellitus in subtotally pancreatectomised rats. Moreover, control by oestrogens of the diabetic state associated with acromegaly has been reported (McCullagh, Beck and Schoffenburg, 1955). The two effects of oestrogens found in our patients, namely regression of malignant disease and return of glucose tolerance to normal, may not be related, since the rise in the increment index in 2 patients who deteriorated on treatment, and in one who failed to show any appreciable change, would suggest that the increment index may usually be expected to rise on treatment with oestrogens, whatever the behaviour of the disease.

The cause of the decrease in glucose tolerance in malignant disease remains a matter for speculation. It would appear unlikely that the carbohydrate metabolism of cancer tissue itself could give rise to this abnormality. Cori and Cori (1925) have shown that tumour tissue *in vivo* has an increased rate of glycolysis. Also the size of tumour in patients in whom this disturbance has been demonstrated is often very small in relation to the total body mass. Suggestions that it may be associated with decreased peripheral utilisation of glucose have not been substantiated (Marks and Bishop, 1957). These authors mention another, more attractive theory, namely that there is an alteration of host tissue metabolism associated with the presence of a neoplastic process. There are reports from animal studies of defects in enzyme activity (Greenstein, 1954) including enzymes involved in carbohydrate metabolism (Weber and Cantero, 1955) of the tissues of the tumour-bearing host. The precise role of oestrogens in altering this abnormality is also obscure.

### SUMMARY

The results of intravenous glucose tolerance test performed on 18 patients with a variety of malignant diseases and on 19 control subjects are presented.

Of the patients with malignant disease, 50 per cent showed diminished glucose tolerance. Where the test was repeated in 12 patients after treatment with oestrogens, glucose tolerance was improved, in six instances from the abnormal to the normal range.

Possible mechanisms and the significance of these results are discussed briefly.

We wish to thank Dr. J. A. Strong for encouragement and guidance in the performance of the study and helpful criticism in the preparation of the paper.

### REFERENCES

AMATUZIO, D. S., STUTZMAN, F. L., VANDERBILT, M. J. AND NESBITT, S.—(1953) *J. clin. Invest.*, **32**, 428.

BENJAMIN, F.—(1960) *Brit. med. J.*, i, 1243.

CORI, C. F. AND CORI, G. T.—(1925) *J. biol. Chem.*, **65**, 397.

DUNCAN, L. P. J.—(1956) *Quart. J. exp. Physiol.*, **41**, 85.

EDWARDS, S.—(1919) *J. Ind. med. Ass.*, **12**, 296.

FREUND, L.—(1885) *Wien. med. Bl.*, **8**, 267.

FRIEDENWOLD, J. AND GROVE, G. H.—(1922) *Amer. J. med. Sci.*, **163**, 33.

GREENSTEIN, J. P.—(1954) ‘ Biochemistry of Cancer ’. 2nd Ed. New York (Academic Press), p. 507.

HOUSSAY, B. A.—(1951) Quoted under “ Annotations ” ; *Lancet*, ii, 70.

KEILIN, D. AND HARTREE, E. F.—(1945) *Biochem. J.*, **39**, 293.—(1948) *Ibid.*, **42**, 230.

McCULLAGH, E. P., BECK, J. C. AND SCHOFFENBURG, C. A.—(1955) *Diabetes*, **4**, 13.

MARKS, P. A. AND BISHOP, J. S.—(1957) *J. clin. Invest.*, **36**, 254.

ROHDENBURG, G. L., BERNHARD, A. AND KREHBIEL, O.—(1919) *J. Amer. med. Ass.*, **72**, 1528.

WEBER, G. AND CANTERO, A.—(1955) *Cancer Res.*, **15**, 105.